# Decoupled molecules with binding polynomials of bidegree (*n*, 2)

**DOI:** 10.1007/s00285-018-1295-x

**Published:** 2018-10-03

**Authors:** Yue Ren, Johannes W. R. Martini, Jacinta Torres

**Affiliations:** 1 Max-Planck-Institut für Mathematik in den Naturwissenschaften, Inselstrasse 22, 04103 Leipzig, Germany; 2 KWS SAAT SE, Grimsehlstrasse 31, 37574 Einbeck, Germany

**Keywords:** Ligand binding, Binding polynomials, Numerical algebraic geometry, 82B05, 92C40, 14Q99, 65H04, 68W30

## Abstract

We present a result on the number of decoupled molecules for systems binding two different types of ligands. In the case of *n* and 2 binding sites respectively, we show that there are $2(n!)^{2}$ decoupled molecules to a generic binding polynomial. For molecules with more binding sites for the second ligand, we provide computational results.

## Introduction

In biology, a ligand is a substance that binds to a target molecule to serve a given purpose. A classical (Bohr et al. 1904; Hasselbalch 1917) and intensively studied (Barcroft 1913; Hill 1913) example is oxygen, which binds reversibly to hemoglobin to be transported through the bloodstream. Reversible mutual binding of different molecules is also a key feature in biological signal transduction (Changeux and Edelstein 2005; Cho et al. 1996; Gutierrez et al. 2009; Ha and Ferrell 2016) and gene regulation (Gutierrez et al. 2012).

A common model for describing equilibrium and steady states of a ligand *L* binding to the sites of a target molecule *M* comes from the grand canonical ensemble of statistical mechanics (Ben-Naim 2001; Hill 1985; Schellman 1975; Wyman and Gill 1990). The grand partition function, in our context also known as the *binding polynomial*, arises as the denominator of the rational function describing the average number of occupied binding sites as a function of ligand activity. In the case of a target molecule with only one binding site, this rational function is given by

$$\begin{aligned} \Psi (\Uplambda ) = \frac{a \Uplambda }{a\Uplambda + 1}, \end{aligned}$$

where the variable $\Uplambda$ denotes the activity of the ligand in the environment, and *a* is a transformation of the binding energy depending on the temperature, which is usually assumed to be constant. This equation is also known as the (sigmoid) Henderson–Hasselbalch titration curve. Titration refers to the laboratory method used to obtain this curve. For systems of molecules with *n* binding sites it generalizes to the Adair equation (Adair et al. 1925; Stefan and Le Novère 2013):

$$\begin{aligned} \Psi (\Uplambda ) = \frac{ n a_n \Uplambda ^n + (n-1)a_{n-1} \Uplambda ^{n-1}+ \cdots + a_1 \Uplambda }{ a_n \Uplambda ^n + a_{n-1} \Uplambda ^{n-1}+ \cdots + a_1 \Uplambda + 1}. \end{aligned}$$

In this model, the binding polynomial and its roots play an important role for the characterization of the binding behavior of the ligand to the target molecule (Briggs 1983, 1984, 1985; Connelly et al. 1986), in particular in the context of cooperativity (Abeliovich 2016; Stefan 2017). The rational functions of systems with *n* binding sites can be represented as sums of *n* Henderson–Hasselbalch curves (Martini and Ullmann 2013; Onufriev et al. 2001; Onufriev and Ullmann 2004), which means that any given system of interacting binding sites can be represented by a hypothetical molecule consisting of stochastic independent binding sites (Martini et al. 2013a) with the same titration curve. The roots of the binding polynomial determine the binding energies of the independent pseudo-sites in this so-called *decoupled sites representation*.

For two different types of ligands, the binding polynomial has two variables (representing the activities of both ligands in the environment) and $(n_1+1) \cdot (m_1+1)$ coefficients $\left( a_{i,j}\right) _{i=0 \ldots n_1;j=0,\ldots ,n_2}$. Given an arbitrary binding polynomial for two types of ligands, it is not in general possible to find a molecule without interactions between *all* binding sites and possessing this binding polynomial. However, molecules can be found in which the binding sites for the same type of ligand do not interact and only interactions between sites for different ligands are non-trivial (Martini et al. 2013b, c). Contrary to the case of one type of ligand, where the decoupled sites representation is unique up to permutation of the roots, there are several different decoupled molecules. It has been shown previously that in the case of *n* and 1 binding sites for the two ligands, respectively, there are *n*! decoupled molecules. The situation becomes more complicated for general systems of $n_1$ and $n_2$ binding sites. The main goal of this paper is to prove the following theorem.

### Theorem 1.1

The decoupled molecules of a fixed binding polynomial of bidegree (*n*, 2) are the solutions to a system of $3n+2$ unknowns: the $n+2$ binding energies and the 2*n* interaction energies. For generic binding polynomials, the number of complex solutions to this system equals $4(n!)^3$. These come in $2(n!)^{2}$ classes under relabeling of the sites.

The article is structured as follows: In Sect. 2, we recall the definition of the binding polynomial and formulate the central question addressed in this work. In Sect. 3, we recall some results and techniques of numerical algebraic geometry, which are necessary to prove the main theorem in Sect. 4. We conclude the article with some experimental results in Sect. 5 and some open questions in Sect. 6.

**Fig. 1:**
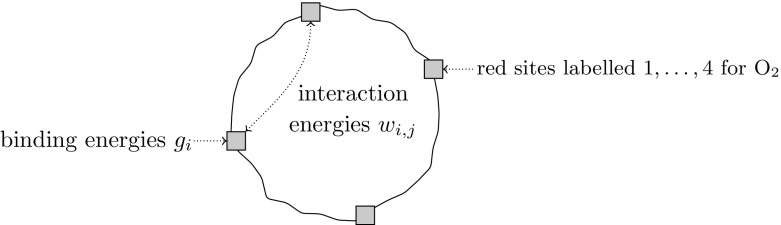
A molecule with 4 binding sites (e.g. hemoglobin)

## Background and framework

In this section, we briefly recap the algebraic framework as well as past results, and, in doing so, fix various notations. Most importantly, we introduce some shorthand notation for molecules with (*n*, 2) sites for Sects. 3 and 4.

### Single type of ligand

The binding behavior of systems with one type of ligand is governed by the energies required to bind to each site of the target molecule and the way different binding sites interact with each other. Following the notation of Martini and Ullmann (2013), we identify target molecules with these parameters.

#### Definition 2.1

A *molecule*$\mathrm{M}$ with *n* sites for one type of ligand is a point

$$\begin{aligned} \mathrm{M}= (g_{1}, \dots , g_{n}, w_{1,2}, w_{1,3}, \dots , w_{n-1,n}) \in ({\mathbb {C}}^*)^n\times ({\mathbb {C}}^{*})^{\left( {\begin{array}{c}n\\ 2\end{array}}\right) }. \end{aligned}$$

The $g_{i}$ are called the *binding energies* and the $w_{i,j}$ are called the *interaction energies*; they measure, respectively, the energy at each site *i* and the interaction energy between sites *i* and *j* (see Fig. 1). We call *Mdecoupled* if $w_{i,j}=1$ for all $1\le i < j\le n$.

We will consider the natural $S_n$ action that corresponds to relabelling the sites:

$$\begin{aligned} \sigma \cdot (g_{1}, \dots , g_{n}, w_{1,2}, \dots , w_{n-1,n}):=(g_{\sigma (1)}, \dots , g_{\sigma (n)}, w_{\sigma (1),\sigma (2)}, \dots , w_{\sigma (n-1),\sigma (n)}) \end{aligned}$$

for $$\sigma \in S_n$$.

#### Definition 2.2

Given a target molecule *M* with *n* sites, we refer to the power set $K:=2^{\{1,\dots ,n\}}$ as the set of all microstates. Each microstate $I \in \mathrm{K}$ describes a binding state by indicating whether binding site *i* is occupied ($i\in I$) or not ($i\notin I$). To each microstate *I* we associate a microstate constant

$$\begin{aligned} a(I) := \prod _{i\in I} \left( g_{i}\prod _{\begin{array}{c} j\in I,\\ j>i\phantom {,} \end{array}}w_{i,j}\right) . \end{aligned}$$

The *binding polynomial* is then defined as

$$\begin{aligned} \mathrm{P}_{M}(\Uplambda ) = \sum _{I \in \mathrm{K}} a(I) \Uplambda ^{|I|} \in \mathbb {C}[\Uplambda ]. \end{aligned}$$

It is a polynomial of degree *n* with constant term 1, and the map $M\mapsto \mathrm{P}_M$ is constant on the $S_n$ orbits, i.e. $\mathrm{P}_{\mathrm{M}}(\Uplambda ) = \mathrm{P}_{\sigma (\mathrm{M})}(\Uplambda )$ for every $\mathrm{M}\in ({\mathbb {C}}^{*})^{\frac{n(n+1)}{2}}$ and all $\sigma \in \mathrm{S}_{n}$.

The following theorem is also known as the *decoupled sites representation*. It implies that any molecule with real binding and interaction energies can be uniquely represented by a molecule with neutral interaction energy, provided that complex binding energies are allowed. Its proof consists of a reformulation of Vieta’s formulas.

#### Theorem 2.3

(Martini and Ullmann 2013, Proposition 2) For any molecule $\mathrm{N}$ there exists a decoupled target molecule $\mathrm{M}$, unique up to relabelling of the sites, such that $\mathrm{P}_{\mathrm{M}} = \mathrm{P}_{\mathrm{N}}$.

### Multiple types of ligands

In case of $d>1$ types of ligands, we consider each binding site to be only able to take up to one type of ligand (Martini et al. 2013c). This is sensible, as we can model a single binding site capable of binding to two types of ligands as two binding sites with interaction energies set so that the two sites can never be saturated at the same time.

For our purposes, let us assume that $d = 2$. We write $n_{1}$ and $n_{2}$ for the number of sites capable of binding to the first and second ligand, respectively.

#### Definition 2.4

**Fig. 2:**
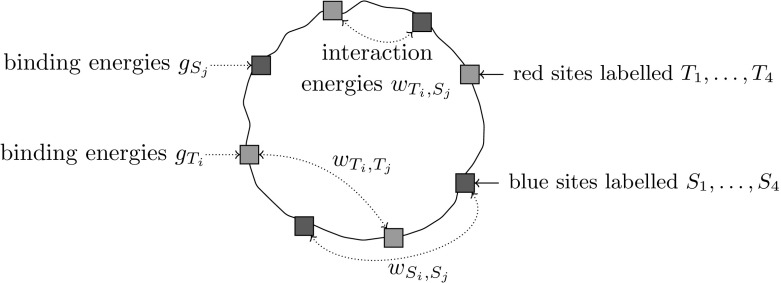
A molecule with (4,4) sites

A *molecule*$\mathrm{M}$ with $(n_1,n_2)$ sites is a point

$$\begin{aligned} \mathrm{M}= (g_{T_1}, \dots , g_{T_{n_1}}, g_{S_1}, \dots , g_{S_{n_2}}, (w_P)_{P\subset \{T_i,S_j\}, |P|=2}) \in ({\mathbb {C}}^*)^{n_1+n_2}\times ({\mathbb {C}}^{*})^{\left( {\begin{array}{c}n_1+n_2\\ 2\end{array}}\right) }, \end{aligned}$$

where $T_1,\dots ,T_{n_1},S_{1},\dots ,S_{n_2}$ represent the binding sites for ligand type *T* and *S* respectively (see Fig. 2) and

- $g_{T_1},\dots ,g_{T_{n_1}}$ and $g_{S_1},\dots ,g_{S_{n_2}}$ are the binding energies,
- $w_P$ for $P\subset \{T_1,\dots ,T_{n_1},S_1,\dots ,S_{n_2}\}$ with $|P|=2$ are the interaction energies.

We call $\mathrm{M}$*decoupled*, if $w_P=1$ for $P\subset \{T_1,\dots ,T_{n_1}\}$ and $P\subset \{S_1,\dots ,S_{n_2}\}$.

Similar to the case $d=1$, there is a natural $S_{n_1}\times S_{n_2}$ action that corresponds to relabelling the sites.

#### Definition 2.5

Similar to the case $d=1$, we can define the binding polynomial $\mathrm{P}_{\mathrm{M}}$ of a molecule $\mathrm{M}$. Explicitly, for decoupled molecules $\mathrm{M}$, $\mathrm{P}_{\mathrm{M}}$ is a bivariate polynomial in the two (ligand) variables $\Uplambda _{1}$ and $\Uplambda _{2}$,

$$\begin{aligned} \mathrm{P}_{\mathrm{M}}(\Uplambda _{1},\Uplambda _{2}) = \sum _{i=1,\dots ,{n_1}} \sum _{j=1,\dots ,n_2} a_{i,j} \Uplambda _{1}^i\Uplambda _{2}^j, \end{aligned}$$

where the coefficients $a_{i,j}$ are given by

1 $$\begin{aligned} a_{i,j} = \underset{|I| = i, \, |J|= j}{\underset{J \subset \{1, \dots , n_{2}\}}{\underset{I \subset \{1, \dots , n_{1}\}}{\sum }}} \;\prod _{T_i\in I} g_{T_i} \prod _{S_j\in J} g_{S_j} \underset{T_i \in J}{\underset{S_j \in I}{\prod }} w_{\{T_i,S_j\}}. \end{aligned}$$

It is a bivariate polynomial of bidegree $(n_1,n_2)$ with constant term 1. Moreover, the map $\mathrm{M}\mapsto \mathrm{P}_\mathrm{M}$ is constant on the $S_{n_1}\times S_{n_2}$-orbits, i.e. $\mathrm{P}_{\mathrm{M}}(\Uplambda ) = \mathrm{P}_{\sigma (\mathrm{M})}(\Uplambda )$ for every $$\mathrm{M}\in (\mathbb C^*)^{n_1+n_2}\times ({\mathbb {C}}^{*})^{\left( {\begin{array}{c}n_1+n_2\\ 2\end{array}}\right) }$$ and all $\sigma \in \mathrm{S}_{n_1}\times \mathrm{S}_{n_2}$.

For $(n_1,n_2)=(n,1)$, the decoupled sites representation takes the following form:

#### Theorem 2.6

(Martini et al. 2013c, Corollary 2) For any molecule $\mathrm{N}$ with (*n*, 1) sites there exist, up to relabelling of the sites, and counted with multiplicity, *n*! decoupled molecules $\mathrm{M}$ of the same type such that ${\mathrm{P}_\mathrm{N}= \mathrm{P}_\mathrm{M}}$.

### Decoupled molecules with (*n*, 2) sites

**Fig. 3:**
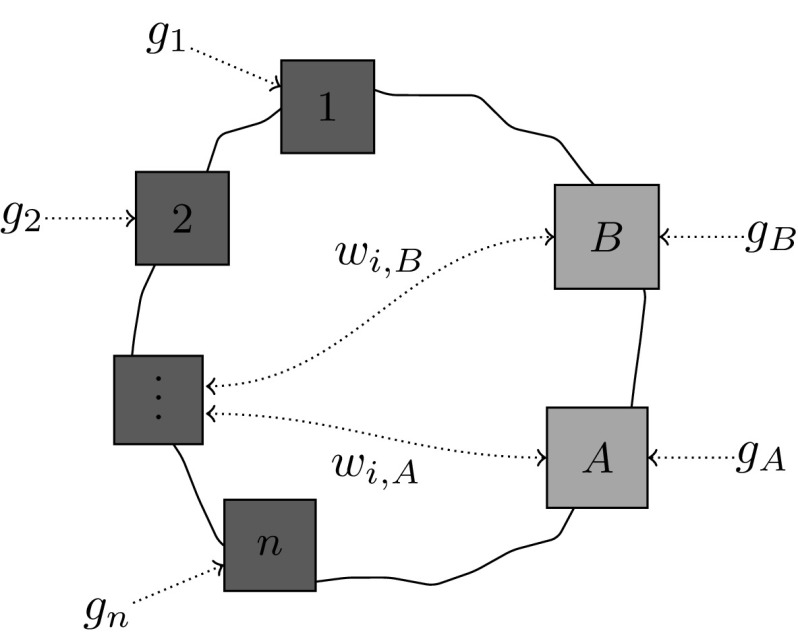
A decoupled molecule with (n,2) sites

The main focus of this article are decoupled molecules with (*n*, 2) sites, for which we will simplify the notation as follows: instead of $T_1,\dots ,T_n$, we label the *n* binding sites of the first type with $1,\dots ,n$, and, instead of $S_1,S_2$, we label the two binding sites of the second type with *A*, *B* (see Fig. 3), so that

- $g_1,\dots ,g_n,g_A,g_B$ represent the binding energies,
- $w_{1,A},\dots ,w_{n,A},w_{1,B},\dots ,w_{n,B}$ represent the non-trivial interaction energies.

**Fig. 4:**
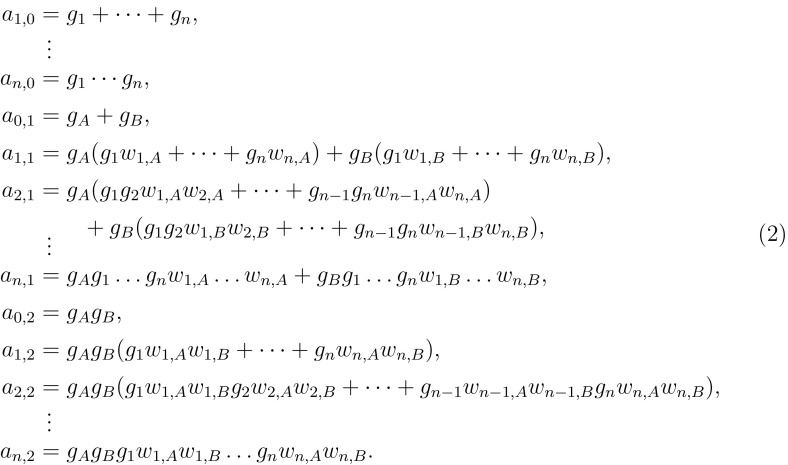
Coefficients of the binding polynomial of bidegree (n,2)

The formulas for the coefficients of the binding polynomial then simplify to the polynomials in System (2) (see Fig. 4). For an explicit instance of the equations and their solutions, see Sect. 5.1. We denote the pair set of decoupled molecules with (*n*, 2) sites and their binding polynomials of bidegree (*n*, 2) (ignoring its constant term 1)

$$\begin{aligned} {\mathcal {M}} = \left\{ ({\underline{g}},{\underline{w}};{\underline{a}})\in ({\mathbb {C}}^*)^{n+2}\times ({\mathbb {C}}^{*})^{n\cdot 2} \times {\mathbb {C}}^{(n+1)\cdot (2+1)-1}\;|\;({\underline{g}},{\underline{w}};{\underline{a}}) \text { satisfies System (2)}\right\} . \end{aligned}$$

## Numerical algebraic geometry

In this section we recall some basic notions of numerical algebraic geometry and its main workhorse: homotopy continuation. For that, we regard ${\mathcal {M}}$ as the preimage of 0 of the polynomial map

$$\begin{aligned} f: \underbrace{({\mathbb {C}}^*)^{n+2}\times ({\mathbb {C}}^{*})^{n\cdot 2}}_{=:X} \times \underbrace{{\mathbb {C}}^{(n+1)\cdot (2+1)-1}}_{=:Y} \rightarrow {\mathbb {C}}^{(n+1)\cdot (2+1)-1} \end{aligned}$$

with

$$\begin{aligned} f({\underline{g}},{\underline{w}}; {\underline{a}}) = \begin{pmatrix} g_1+\dots +g_n - a_{1,0} \\ \vdots \\ \vdots \\ \end{pmatrix}, \end{aligned}$$

where ${\underline{g}}:=(g_1,\dots ,g_n,g_A,g_B)$ and $\underline{w}:=(w_{1,A},\dots ,w_{n,A},w_{1,B},\dots ,w_{n,B})$ are referred to as *unknowns*, and ${\underline{a}}=(a_{1,0},\dots ,a_{n,2})$ are regarded as *parameters*. We fix the projection

$$\begin{aligned} \pi _Y : X\times Y\longrightarrow Y. \end{aligned}$$

One fundamental and important concept is that solutions vary continuously in the parameters, which is summarized in the following theorem.

### Theorem 3.1

(Sommese and Wampler 2005, Theorem A.14.1) If there is an isolated solution $({\underline{g}}^*,\underline{w}^*;{\underline{a}}^*)\in X\times Y$ of $f({\underline{g}},\underline{w};{\underline{a}}^*)=0$, then there are euclidean open sets $\mathcal U\subset X$, ${\mathcal {V}} \subset Y$ containing $(\underline{g}^*,{\underline{w}}^*)$, ${\underline{a}}^*$ respectively such that

1. $({\underline{g}}^*,{\underline{w}}^*)$ is the only solution of $f({\underline{g}},{\underline{w}};{\underline{a}}^*)=0$ for $({\underline{g}},{\underline{w}})\in {\mathcal {U}}$;
2. for fixed $a'\in {\mathcal {V}}$, $f({\underline{g}},{\underline{w}};{\underline{a}}')=0$ has only isolated solutions for $({\underline{g}},{\underline{w}})\in {\mathcal {U}}$;
3. for fixed $a'\in {\mathcal {V}}$, the multiplicity of $({\underline{g}}^*,{\underline{w}}^*;{\underline{a}}^*)$ as a solution of $f({\underline{g}},{\underline{w}};{\underline{a}}^*)=0$ is the sum of the multiplicities of the solutions of $f({\underline{g}},{\underline{w}};{\underline{a}}')=0$ for $({\underline{g}},{\underline{w}})\in {\mathcal {U}}$.

### Example 3.2

Consider the first *n* components of our polynomial map, which are given by (abbreviating $a_i:=a_{i,0}$):

$$\begin{aligned} f(g_1,\dots ,g_n;a_1,\dots ,a_n) := \begin{pmatrix} g_1+\dots +g_n-a_1 \\ g_1g_2 + g_1g_3 + \dots + g_{n-1}g_n-a_2 \\ \vdots \\ g_1 \cdots g_n - a_n \end{pmatrix}. \end{aligned}$$

Given any parameter ${\underline{a}}'\in \mathbb {C}^n$, one can show that any solution ${\underline{g}}'\in \mathbb {C}^n$ to $f({\underline{g}};\underline{a}')=0$ consists of the roots of the univariate polynomial $t^n+a_1' t^{n-1} + \dots + a_n'$. This is commonly known as Vieta’s formula (Hazewinkel 2013). Hence there exist a Zariski-open set ${\mathcal {U}}:= \mathbb {C}^n \setminus \text {Disc}_x(x^n+a_1 x^{n-1} + \dots + a_n)$ such that for any ${\underline{a}}'\in {\mathcal {U}}$ there are *n*! distinct simple solutions to $f({\underline{g}};{\underline{a}}')=0$. We say that there are *genericallyn*! solutions and refer to $\underline{a}'\in {\mathcal {U}}$ as a *generic* choice of parameters.

Should $x^n+a_1' x^{n-1} + \dots + a_n'=(x-1)^n$, then the only solution is ${\underline{g}}'=(1,\dots ,1)$. Theorem 3.1 implies that this solution is of multiplicity *n*!. This will be important in the proof of Lemma 4.2.

The arguably most essential tool in numerical algebraic geometry is path tracking: Given

- a starting solution $$(g',w';a')\in X\times Y$$,
- a target parameter $$a^*\in Y$$,
- a continuous path $\phi :[0,1]\rightarrow Y$ with $\phi (1)=a'$ and $\phi (0)=a^*$,

there exist under good circumstances (Sommese and Wampler 2005, Theorem 7.1.6) a *solution path*

$$\begin{aligned} z: (0,1] \rightarrow X \text { with } z(1)=(g',w') \text { and } f(z(t),\phi (t))=0. \end{aligned}$$

However, the solution path might diverge, which is why these problems are commonly studied in projective space.

### Example 3.3

**Fig. 5:**
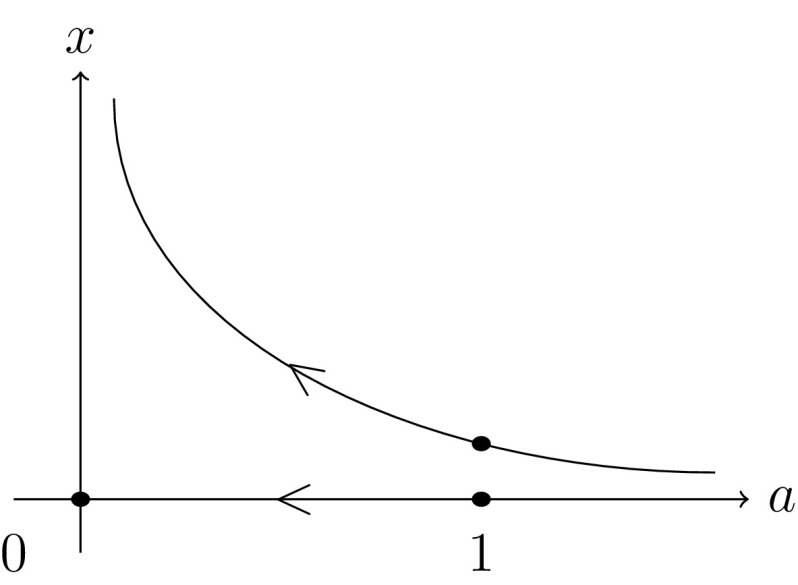
A converging and a diverging solution path

(solutions at infinity) The simplest example of diverging solution path is the function

$$\begin{aligned} f: \mathbb {C}\times \mathbb {C}\longrightarrow \mathbb {C},\quad f(x;a)=ax^2-x, \end{aligned}$$

with two starting solutions (0; 1), (1; 1), the target parameter 0 and the continuous, straight-line path $\phi : [0,1] \rightarrow \mathbb {C}, t\mapsto 1-t$, see Fig. 5.

The two solution paths are

$$\begin{aligned} z_1:&\;\; [0,1) \longrightarrow \mathbb {C}, \quad t\longmapsto 0, \\ z_2:&\;\; [0,1) \longrightarrow \mathbb {C}, \quad t\longmapsto \frac{1}{1 - t }, \end{aligned}$$

of which the first obviously converges, while the second diverges. Note that diverging paths can only appear if parameters occur in the coefficients of non-constant monomials, which is not the case in System (2), see proof of Theorem 4.4.

## Generic decoupled molecules with (*n*, 2) sites

In this section, we show that a generic binding polynomial represents $4\cdot (n!)^3$ decoupled molecules with (*n*, 2) sites. Due to the complexity of the system of polynomial equations, the proof is split in two parts. First, we study a special class of decoupled molecules and their binding polynomials. In a second step, we study their implication to the generic case.

### Normalized molecules

In this subsection, we restrict ourselves to a special class of decoupled molecules and their binding polynomials. This simplifies our system of equations and allows us to show that a generic binding polynomial of such molecules represents 2*n*! molecules, each of multiplicity $2(n!)^{2}$.

#### Definition 4.1

Recall that ${\mathcal {M}}$ consists pairs of molecules and their binding polynomials (see Sect. 2.3). We define the set of all *normalized* molecules to be

$$\begin{aligned} {\mathcal {M}}_{\text {norm}}:=\left\{ ({\underline{g}},{\underline{w}};{\underline{a}})\in {\mathcal {M}}\;\Bigg \vert \begin{array}{c} g_i=1 \text { for } i=1,\dots ,n \text { and } g_A=g_B=1 \\ w_{i,A} w_{i,B}=1 \text { for } i=1,\dots ,n \end{array} \right\} . \end{aligned}$$

#### Lemma 4.2

The projection

$$\begin{aligned} {\mathcal {M}}{\mathop {\longrightarrow }\limits ^{\pi _{\text {norm}}}}(\mathbb {C}^*)^n\times \mathbb {C}^n, \quad ({\underline{g}},{\underline{w}};{\underline{a}})\longmapsto (w_{1,A},\dots ,w_{n,A};a_{1,1},\dots ,a_{n,1}) \end{aligned}$$

maps ${\mathcal {M}}_{\text {norm}}$ bijectively onto the affine variety *V* cut out by

3 $$\begin{aligned} \begin{aligned} a_{1,1}&= (w_{1,A}+\dots +w_{n,A})+ \left( \frac{1}{w_{1,A}}+\dots +\frac{1}{w_{n,A}}\right) ,\\&\;\,\vdots \\ a_{n,1}&= w_{1,A}\dots w_{n,A} + \frac{1}{w_{1,A}\dots w_{n,A}}. \end{aligned} \end{aligned}$$

Moreover, ${{\mathcal {M}}}$ has multiplicity $2(n!)^2$ along ${\mathcal M}_{norm}$.

#### Proof

The bijection follows directly from the conditions on $\mathcal M_{\text {norm}}$ and the equations of System (2): If $({\underline{g}},{\underline{w}};{\underline{a}})$ is normalized, then by definition ${\underline{g}}=(1,\dots ,1)$ and $w_{1,B} = w_{1,A}^{-1}$. Additionally, the following parameters are uniquely determined by the following equations of System (2):

$$\begin{aligned} a_{0,1}&= g_A + g_B,&a_{0,2}&= g_Ag_B, \\ a_{1,0}&= g_1+\dots +g_n,&a_{1,2}&= g_Ag_B (g_1 w_{1,A}w_{1,B}+\dots +g_n w_{n,A}w_{n,B}),\\&\;\,\vdots&\;\,\vdots \\ a_{n,0}&= g_1\cdots g_n,&a_{n,2}&= g_Ag_B g_1 w_{1,A}w_{1,B}\dots g_n w_{n,A}w_{n,B}. \end{aligned}$$

The multiplicity follows from the fact that:

- any solution $(g_A,g_B;a_{0,j})$ to the two equations in the first row is of multiplicity 2 (see Example 3.2),
- the solution $$(g_1,\dots ,g_n;a_{i,0})=(1,\dots ,1;\left( {\begin{array}{c}n\\ i\end{array}}\right) )$$ to the latter equations in the first column is of multiplicity *n*!,
- given $g_A=g_B=g_i=1$, the solution $$(w_{i,A},w_{i,B};a_{i,2})=(1,\dots ,1;\left( {\begin{array}{c}n\\ i\end{array}}\right) )$$ to the latter equations in the second column is of multiplicity *n*!.

and from the fact that the multiplicity of the entire system equals the product of the multiplicities of the three smaller systems in our case (Eisenbud and Harris 2016, Proposition 1.29). $\square$

#### Proposition 4.3

A generic normalized binding polynomial represents 2*n*! decoupled molecules, each of multiplicity $2(n!)^{2}$.

#### Proof

By Lemma 4.2, it suffices to show that System (3) has 2*n*! simple solutions for generic ${\underline{a}} = (a_{1,1},\dots ,a_{n,1})\in \mathbb {C}^n$, or rather for $(a_{1,1},\dots ,a_{n,1})\in {\mathcal {U}}$ for some Euclidean open subset ${\mathcal {U}}\subseteq \mathbb {C}^n$. For the sake of simplicity, we abbreviate $a_i:= a_{i,1}$ and $w_i:=w_{i,A}$ for $i=1,\dots ,n$. Next, we introduce *n* new variables $\mu _1,\dots ,\mu _n$ and consider the following equivalent system of 2*n* equations in the 2*n* variables $\mu _1,\dots ,\mu _n,w_1\dots ,w_n$: 
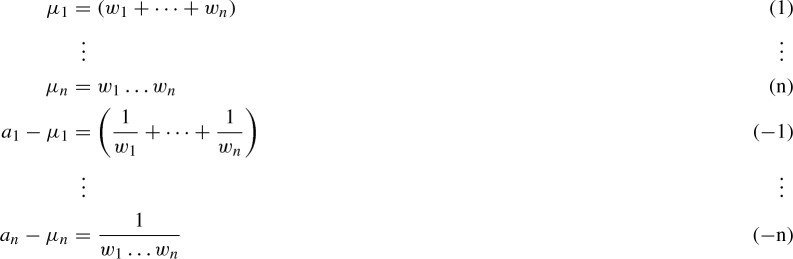


Let ${\mathcal {N}}$ be the variety cut out by the system above and let $\pi _\mu$ and $\pi _a$ denote the three projections onto $\mu _i$ and $a_i$ respectively.
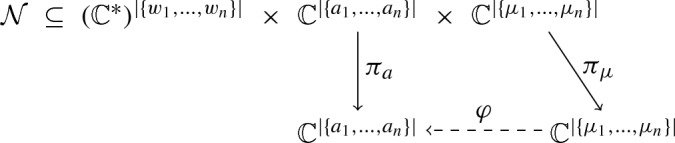


We will construct a dominant (i.e. its image is Zariski dense), 2:1 rational map

$$\begin{aligned} \varphi : \mathbb {C}^n \dashrightarrow \mathbb {C}^n, \quad (\mu _1,\dots ,\mu _n) \longmapsto (a_1,\dots ,a_n), \end{aligned}$$

that maps ${\underline{\mu }}$ to the unique ${\underline{a}}$ for which some ${\underline{w}}$ exists such that $({\underline{w}};\underline{a},{\underline{\mu }})\in {\mathcal {N}}$. In short, the diagram above commutes. The image of the complement $\mathbb {C}^n\setminus \text {Disc}_x(x^n-\mu _1 x^{n-1} + \dots + (-1)^n \mu _n)$ will then contain an open set ${\mathcal {U}}\subseteq \mathbb {C}^n$, and for any ${\underline{a}}\in {\mathcal {U}}$ the system will have 2*n*! solutions: 2 solutions in ${\underline{\mu }}$, both outside the discriminant, and consequently also *n*! solutions in ${\underline{w}}$ for each ${\underline{\mu }}$.

To construct $\varphi$, observe that combining equations $(-n)$ and (*n*) and obtain:

$$\begin{aligned} a_n -\mu _n = \frac{1}{w_1\dots w_n} = \frac{1}{\mu _n}, \end{aligned}$$

which is equivalent to

$$\begin{aligned} \mu _n^2-a_n\cdot \mu _n+1 = 0 \quad \text {or}\quad a_n=\mu _n+\frac{1}{\mu _n}. \end{aligned}$$

Moreover, multiplying equation $(-(n-1))$ with $x_1\dots x_n$ yields

$$\begin{aligned} (a_{n-1}-\mu _{n-1})\cdot \underbrace{x_1\dots x_n}_{\overset{\text {Eq.}(n)}{=} \mu _n} = \underbrace{x_1+\dots +x_n}_{\overset{\text {Eq.}(1)}{=} \mu _1}, \end{aligned}$$

or, more generally, by multiplying Equation $(-i)$ with $x_1\dots x_n$:

$$\begin{aligned} (a_i-\mu _i)\cdot \mu _n = \mu _{n-i} \quad \text {or}\quad a_i = \mu _i - \frac{\mu _{n-i}}{\mu _n}\quad \text {for } i=1,\dots ,n-1. \end{aligned}$$

Set

$$\begin{aligned} \varphi : \mathbb {C}^n&\dashrightarrow \mathbb {C}^n,\\ (\mu _1,\dots ,\mu _n)&\longmapsto \left( \mu _1-\frac{\mu _{n-1}}{\mu _n},\dots ,\mu _{n-1}-\frac{\mu _1}{\mu _n},\mu _n-\frac{1}{\mu _n}\right) \end{aligned}$$

By construction, $\varphi$ is 2 : 1 and commutes with the projections $\pi _\mu$, $\pi _a$. Moreover, it is dominant as its Jacobian,

$$\begin{aligned} J(\phi ) = \begin{pmatrix} 1 &{} &{} &{} &{} -1 \\ &{} 1 &{} &{} -1 \\ &{} &{} \ddots \\ &{} -1 &{} &{} 1 \\ \frac{\mu _{n-1}}{\mu _n^2} &{} \frac{\mu _{n-2}}{\mu _n^2} &{} \dots &{} \frac{\mu _{1}}{\mu _n^2} &{} 1+ \frac{1}{\mu _n^2} \end{pmatrix} \end{aligned}$$

is invertible at $(1,\dots ,1)$. $\square$

### A generic decoupled sites representation

In this subsection, we will infer from Proposition 4.3 the number of molecules a generic binding polynomial represents.

**Fig. 6:**
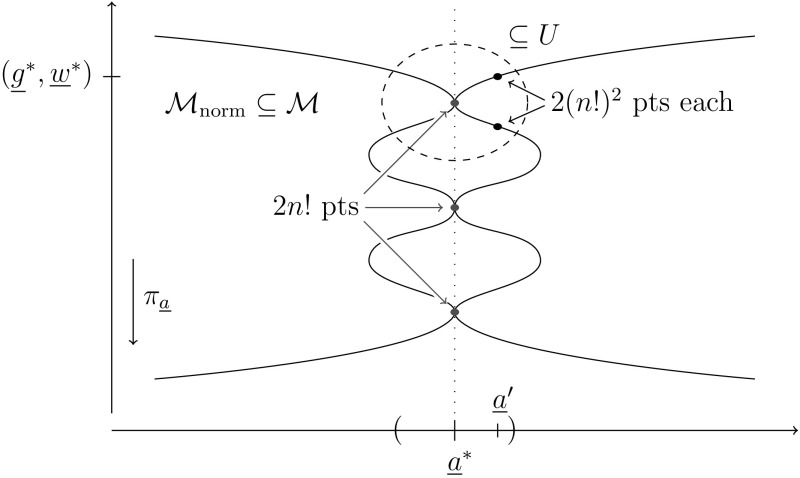
Perturbation of normalized binding polynomials

#### Theorem 4.4

A generic binding polynomial of bidegree (*n*, 2) represents $4(n!)^3$ decoupled molecules with (*n*, 2) sites. These come in $2(n!)^2$ classes modulo the $S_n\times S_2$ action that corresponds to relabelling of the sites.

#### Proof

Similar to the proof of Proposition 4.3, it suffices to show the claim in a euclidean open set. For that consider a generic normalized binding polynomial ${\underline{a}}^*\in \mathbb {C}^{(n+1)(2+1)-1}$. Proposition 4.3 states that there are 2*n*! solutions $({\underline{g}}^*,\underline{w}^*;{\underline{a}}^*)$ to $f({\underline{g}},{\underline{w}};\underline{a}^*)=0$ of multiplicity $2(n!)^2$, and we will now argue why each of them yields $2(n!)^2$ solutions if we perturb ${\underline{a}}^*$ slightly, see Fig. 6.

Applying Theorem 3.1 to each of the solutions, we obtain an open subset $U\subseteq X\times Y$, where $X:=({\mathbb {C}}^*)^{n+2} \times ({\mathbb {C}}^*)^{n\cdot 2}$, $Y:={\mathbb {C}}^{3n+2}$, such that

- for any ${\underline{a}}'\in \pi _{{\underline{a}}}(U)$, $U\cap X \times \{a'\})$ has only isolated solutions of $f({\underline{g}}, {\underline{w}};{\underline{a}}')=0$,
- the sum of the multiplicities of those isolated solutions is $4(n!)^3$.

It remains to show that *U* contains all isolated solutions of $f({\underline{g}}, {\underline{w}};{\underline{a}}')=0$ for $\underline{a}'\in \pi _{{\underline{a}}}(U)$ and that the solutions are simple. Both follow from the fact that our parameters are exactly the constant terms, i.e. we can regard ${\mathcal {M}}$ as the graph of the polynomial map

$$\begin{aligned} h:X \longrightarrow Y, \quad ({\underline{g}},{\underline{w}}) \longmapsto \begin{pmatrix} g_1+\dots +g_n \\ \vdots \\ \vdots \end{pmatrix}, \end{aligned}$$

so that $f({\underline{g}},{\underline{w}};{\underline{a}})=0$ is equivalent to $h({\underline{g}},{\underline{w}})={\underline{a}}$. Fix $a'\in \pi _{{\underline{a}}}(U)$ and a path

$$\begin{aligned} \phi :[0,1]\rightarrow Y,\quad \phi (0) = a^* \text { and } \phi (1) = a'. \end{aligned}$$

To see that *U* contains all solutions to $f({\underline{g}}, {\underline{w}};{\underline{a}}')=0$, observe that any solution $(g',w';a')$ has a solution path

$$\begin{aligned} z:(0,1]\longrightarrow X \text { with } z(1)=(g',w') \end{aligned}$$

such that $f(z(t);\phi (t))=0$ for all $t\in (0,1]$. As $\lim _{t\rightarrow 0} h(z(t)) = \lim _{t\rightarrow 0} \phi (t) = {\underline{a}}^*$ and *h* is polynomial, $\lim _{t\rightarrow 0} z(t)$ has to converge. Therefore $(\lim _{t\rightarrow 0} z(t),{\underline{a}}^*)$ is one of our 2*n*! solutions, which implies $(g',w';a')\in U$.

To see that solutions are simple for generic binding polynomial note that a solution $(g',w';a')$ to $f({\underline{g}},\underline{w};{\underline{a}}')=0$ is singular if and only if the point $(g',w')$ is a critical point of *h*. Hence, any solution in the following open set will be simple

$$\begin{aligned} {\mathcal {U}} := U\cap \pi _{{\underline{a}}}^{-1}(Y\setminus S), \end{aligned}$$

where $S:= \{ h(g',w')\in \mathbb {C}^{\{a_{i,j}\}} \mid (g',w') \text { critical point}\}$ consists of the images of all critical points of *h*. It remains to show that that *S* is not the entire ambient space *Y*, so that the set of all critical points of *h* is a proper subvariety of *X* of positive codimension and so is the Zariski-closure of *S*. This is equivalent to the fact that the Jacobian of *h* has a non-zero determinant, which we will show defining an ordering on the set of all monomials and proving that the determinant has a non-zero maximal monomial with respect to it.

Let > be the monomial ordering defined by

$$\begin{aligned}&g_A^{\alpha _A} g_B^{\alpha _B} g_1^{\alpha _1}\cdots g_n^{\alpha _n} w_{1,A}^{\alpha _{1,A}} \cdots w_{n,B}^{\alpha _{n,B}}> g_A^{\beta _A} g_B^{\beta _B} g_1^{\beta _1}\cdots g_n^{\beta _n} w_{1,A}^{\beta _{1,A}} \cdots w_{n,B}^{\beta _{n,B}}\\&\quad \Longleftrightarrow \quad \Big (\alpha _w:=(\alpha _{1,A}, \dots , \alpha _{n,B})>_{\text {lex}} \beta _w:=(\beta _{1,A}, \dots , \beta _{n,B})\Big ) \text { or}\\&\qquad \Big (\alpha _w=\beta _w \text { and } (\alpha _A,\alpha _B,\alpha _1,\dots ,\alpha _n) >_{\text {lex}} (\beta _A,\beta _B,\beta _1,\dots ,\beta _n)\Big ), \end{aligned}$$

where $>_\text {lex}$ is the lexicographical ordering on $\mathbb {N}^k$ for arbitrary *k*, i.e.

$$\begin{aligned}&(\alpha _1,\dots ,\alpha _k)> (\beta _1,\dots ,\beta _k) \\&\quad \Longleftrightarrow \quad \alpha _1=\beta _1, \ldots , \alpha _{i-1}=\beta _{i-1} \text { and } \alpha _{i}>\beta _i \text { for some } 0<i<k. \end{aligned}$$

Letting $h_{i,j}$ denote the component of *h* consisting of the polynomial on the right-hand-side of $a_{i,j}$ in System (2), the Jacobian of *h* is of the form

$$\begin{aligned} \left( \begin{array}{c|c} J_g &{} 0 \\ \hline *&{} J_w \end{array} \right) ,&\text { where } J_g := \begin{pmatrix} \partial _{g_A} h_{0,1} &{} \partial _{g_B} h_{0,1} \\ \partial _{g_A} h_{0,2} &{} \partial _{g_B} h_{0,2} \\ &{} &{} \partial _{g_1} h_{1,0} &{} \ldots &{} \partial _{g_n} h_{1,0} \\ &{} &{} \vdots &{} &{} \vdots \\ &{} &{} \partial _{g_1} h_{n,0} &{} \ldots &{} \partial _{g_n} h_{n,0} \\ \end{pmatrix} \\&\text { and } \;\;\, J_w := \begin{pmatrix} \partial _{w_{1,A}} h_{1,1} &{} \ldots &{} \partial _{w_{n,A}} h_{1,1} &{} \partial _{w_{1,B}} h_{1,1} &{} \ldots &{} \partial _{w_{n,B}} h_{1,1} \\ \vdots &{} &{} \vdots &{} \vdots &{} &{} \vdots \\ \partial _{w_{1,A}} h_{n,1} &{} \ldots &{} \partial _{w_{n,A}} h_{n,1} &{} \partial _{w_{1,B}} h_{n,1} &{} \ldots &{} \partial _{w_{n,B}} h_{n,1} \\ \partial _{w_{1,A}} h_{1,2} &{} \ldots &{} \partial _{w_{n,A}} h_{1,2} &{} \partial _{w_{1,B}} h_{1,2} &{} \ldots &{} \partial _{w_{n,B}} h_{1,2} \\ \vdots &{} &{} \vdots &{} \vdots &{} &{} \vdots \\ \partial _{w_{1,A}} h_{n,2} &{} \ldots &{} \partial _{w_{n,A}} h_{n,2} &{} \partial _{w_{1,B}} h_{n,2} &{} \ldots &{} \partial _{w_{n,B}} h_{n,2} \\ \end{pmatrix}, \end{aligned}$$

and it remains to show that both $J_g$ and $J_w$ have non-zero determinant.

Explicitly, $J_g$ is of the form

$$\begin{aligned} \begin{pmatrix} 1 &{} 1 \\ g_B &{} g_A \\ &{} &{} 1 &{} 1 &{} \ldots &{} 1 \\ &{} &{} \displaystyle \sum _{i_1\ne 1} g_{i_1}&{} \displaystyle \sum _{i_1\ne 2} g_{i_1} &{}\ldots &{} \displaystyle \sum _{i_1\ne n} g_{i_1} \\ &{} &{} \displaystyle \sum _{\begin{array}{c} i_1,i_2\ne 1\\ i_1<i_2 \end{array}} g_{i_1}g_{i_2}&{} \displaystyle \sum _{\begin{array}{c} i_1,i_2\ne 2\\ i_1<i_2 \end{array}} g_{i_1}g_{i_2} &{}\ldots &{} \displaystyle \sum _{\begin{array}{c} i_1,i_2\ne n\\ i_1<i_2 \end{array}} g_{i_1}g_{i_2} \\ &{} &{} \vdots &{} \vdots &{} &{} \vdots \\ &{} &{} \prod _{j\ne 1} g_{j}&{} \prod _{j\ne 2} g_{j} &{}\ldots &{} \prod _{j\ne n} g_{j} \\ \end{pmatrix}. \end{aligned}$$

The following monomial, which is contained in the product of all diagonal entries, is maximal among the monomial appearing in the Leibniz formula for determinants:

$$\begin{aligned} s_g:=(1)\cdot (g_A) \cdot \underbrace{(1)}_{s_3} \cdot \underbrace{(g_1)}_{s_4} \cdot \underbrace{(g_1 g_2)}_{s_5} \cdots \underbrace{(g_1\cdots g_{n-1})}_{s_{n+2}} \end{aligned}$$

Moreover, this monomial can only be obtained by multiplying the diagonal elements, because each $s_i$ maximal in its row and and the earlier entries consists of monomials strictly smaller than it. This implies that $s_g$ occurs only once in the Leibniz formula for determinants, making it the maximal monomial in the thus non-zero determinant.

Ignoring $g_A,g_B,g_1,\ldots ,g_n$, which can be ignored in the search of the largest monomial due to our choice of ordering, and abbreviating $w_{i,AB}:=w_{i,A}w_{i,B}$, the matrix $J_w$ is of the form:

$$\begin{aligned} \begin{pmatrix} 1 &{} \dots &{} 1 &{} 1 &{} \dots &{} 1 \\ \displaystyle \sum _{i_1\ne 1} w_{i_1,A} &{} \dots &{} \displaystyle \sum _{i_1\ne n} w_{i_1,A} &{} \displaystyle \sum _{i_1\ne 1} w_{i_1,B} &{} \dots &{} \displaystyle \sum _{i_1\ne n} w_{i_1,B} \\ \vdots &{} &{} \vdots &{} \vdots &{} &{} \vdots \\ \displaystyle \prod _{j\ne 1} w_{j,A} &{} \dots &{} \displaystyle \prod _{j\ne n} w_{j,A} &{} \displaystyle \prod _{j\ne 1} w_{j,B} &{} \dots &{} \displaystyle \prod _{j\ne n} w_{j,B} \\ w_{1,B} &{} \dots &{} w_{n,B} &{} w_{1,A} &{} \dots &{} w_{n,A} \\ w_{1,B} \displaystyle \sum _{i_1\ne 1} w_{i_1,AB} &{} \dots &{} w_{n,B} \displaystyle \sum _{i_1\ne n} w_{i_1,AB} &{} w_{1,A} \displaystyle \sum _{i_1\ne 1} w_{i_1,AB} &{} \dots &{} w_{n,A} \displaystyle \sum _{i_1\ne n} w_{i_1,AB} \\ \vdots &{} &{} \vdots &{} \vdots &{} &{} \vdots \\ w_{1,B} \displaystyle \prod _{j\ne 1} w_{j,AB} &{} \dots &{} w_{n,B} \displaystyle \prod _{j\ne n} w_{j,AB} &{} w_{1,A} \displaystyle \prod _{j\ne 1} w_{j,AB} &{} \dots &{} w_{n,A} \displaystyle \prod _{j\ne n} w_{j,AB} \end{pmatrix}. \end{aligned}$$

The following monomial, contained in the product of all diagonal entries, is maximal among the monomials appearing in the Leibniz formula for determinants:

$$\begin{aligned}&s_w:=(1)\cdot (w_{1,A}) \cdots (w_{1,A}\cdots w_{n-1,A}) \cdot (w_{1,A}) \cdot \\&\quad (w_{1,A} w_{2,AB}) \cdots (w_{1,A} w_{2,AB} \cdots w_{n-1,AB}) \end{aligned}$$

As before, this polynomial can only be obtained by multiplying all diagonal entries, as the earlier entries in a row consists of strictly smaller monomials. This implies that $s_w$ occurs only once in the Leibniz formula for determinants, making it the maximal monomial in the thus non-zero determinant. $\square$

## Further experimental results

In this section, we provide some experimental results for (*n*, 2) and beyond. For simplicity, we will use randomly chosen $\underline{a} \in \mathbb {C}^{(n+1)(2+1)-1}$. Moreover, we will also fix a choice of $g_{S_1},\dots ,g_{S_{n_1}},g_{T_1},\dots ,g_{T_{n_2}}$ to factor out the natural $S_{n_1}\times S_{n_2}$ action on the roots of System (1), see Sect. 5.1.

All computations are done using one of the following three programs:

- bertini (https://bertini.nd.edu/): A solver for polynomial equations using numerical algebraic geometry. It has built-in features for parallel path-tracking, which proved to be particularly useful for big examples.
- gfan (http://home.math.au.dk/jensen/software/gfan/gfan.html): A software package for computing Gröbner fans and tropical varieties. It features a new algorithm for computing mixed volumes using tropical homotopy methods (Jensen 2016).
- Singular (Decker et al. 2016): A computer algebra system for polynomial computations, with special emphasis on commutative and non-commutative algebra, algebraic geometry, and singularity theory.

Scripts, tutorials and other auxiliary files for the computations are available at https://software.mis.mpg.de.

### Explicit solutions for (3, 2)

Consider the following equations of System (2) for $n=3$:

$$\begin{aligned} a_{1,1}&= g_A (g_1w_{1,A}+g_2w_{2,A}+g_3w_{3,A})+ g_B (g_1w_{1,B}+g_2w_{2,B}+g_nw_{3,B}),\\ a_{2,1}&= g_A (g_1g_2w_{1,A}w_{2,A}+g_1g_3w_{1,A}w_{3,A}+g_2g_3w_{2,A}w_{3,A})\\&\qquad +g_B (g_1g_2w_{1,B}w_{2,B}+g_1g_3w_{1,B}w_{3,B}+g_2g_3w_{2,B}w_{3,B}), \\ a_{3,1}&= g_A g_1 g_2 g_3 w_{1,A} w_{2,A} w_{3,A} + g_B g_1 g_2 g_3 w_{1,B} w_{2,B} w_{3,B},\\ a_{1,2}&= g_Ag_B (g_1 w_{1,A}w_{1,B}+ g_2 w_{2,A}w_{2,B} +g_3 w_{3,A}w_{3,B}),\\ a_{2,2}&= g_Ag_B (g_1 g_2 w_{1,A}w_{1,B} w_{2,A}w_{2,B}+ g_1 g_3 w_{1,A}w_{1,B} w_{3,A}w_{3,B} \\&\quad + g_2 g_3 w_{2,A}w_{2,B} w_{3,A}w_{3,B}), \\ a_{3,2}&= g_Ag_B g_1 g_2 g_3 w_{1,A}w_{1,B}w_{2,A}w_{2,B} w_{3,A}w_{3,B}. \end{aligned}$$

Choosing

$$\begin{aligned} g_1&= 2, \quad g_2 = 3, \quad g_3 = 5, \quad g_A = 11, \quad g_B = 13, \\ a_{1,1}&= 71, \quad a_{2,1}=73, \quad a_{3,1}=79, \quad a_{1,2}=101,\quad a_{2,2}=103, \quad a_{3,2}=107, \end{aligned}$$

the system then simplifies to

$$\begin{aligned} 71&= 11 (2w_{1,A}+3w_{2,A}+5w_{3,A})+ 13 (2w_{1,B}+3w_{2,B}+5w_{3,B}),\\ 73&= 11 (6w_{1,A}w_{2,A}+10w_{1,A}w_{3,A}+15w_{2,A}w_{3,A})\\&\qquad +13 (6w_{1,B}w_{2,B}+10w_{1,B}w_{3,B}+15w_{2,B}w_{3,B}), \\ 79&= 330 w_{1,A} w_{2,A} w_{3,A} + 390 w_{1,B} w_{2,B} w_{3,B},\\ 101&= 143 (2 w_{1,A}w_{1,B}+ 3 w_{2,A}w_{2,B} +5 w_{3,A}w_{3,B}),\\ 103&= 143 (6 w_{1,A}w_{1,B} w_{2,A}w_{2,B}+ 10 w_{1,A}w_{1,B} w_{3,A}w_{3,B}\\&\quad + 15 w_{2,A}w_{2,B} w_{3,A}w_{3,B}), \\ 107&= 4290 w_{1,A}w_{1,B}w_{2,A}w_{2,B} w_{3,A}w_{3,B}. \end{aligned}$$

**Fig. 7:**
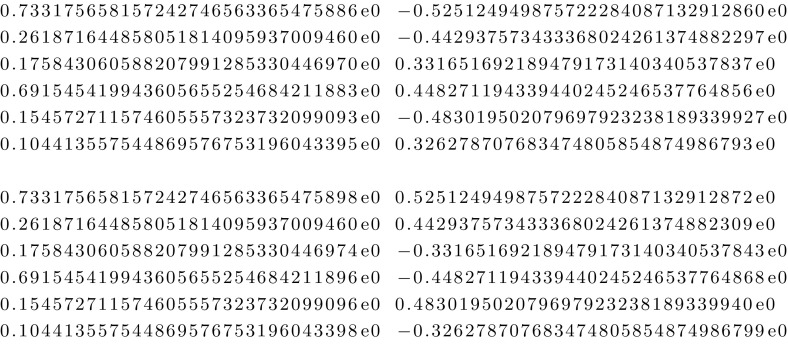
Two complex conjugate solutions for (3, 2)

Using bertini, we see that it has 72 roots, all of which are non-real and simple, 12 of which have strictly positive real component. Figure 7 shows a pair of complex conjugate solutions with lexicographically largest real part. The ordering on the variables is $w_{1,A}$, $w_{1,B}$, $w_{2,A}$, $w_{2,B}$, $w_{3,A}$, $w_{3,B}$, so that the first lines corresponds to the real and imaginary part of $w_{1,A}$.

### Mixed volumes

The Newton polytope of a polynomial is the convex hull of all exponent vectors of all monomials with non-zero coefficients. Given a polynomial system $f_1,\dots ,f_N$ in *N* variables and with only finitely many solutions, the *mixed volume* of their Newton polytopes is an upper bound on the number of solutions that is attained provided the non-zero coefficients are generic. This is known as the Bernstein–Khovanskii–Kushnirenko Theorem (Bernstein 1975), or BKK Theorem in short.

**Fig. 8:**
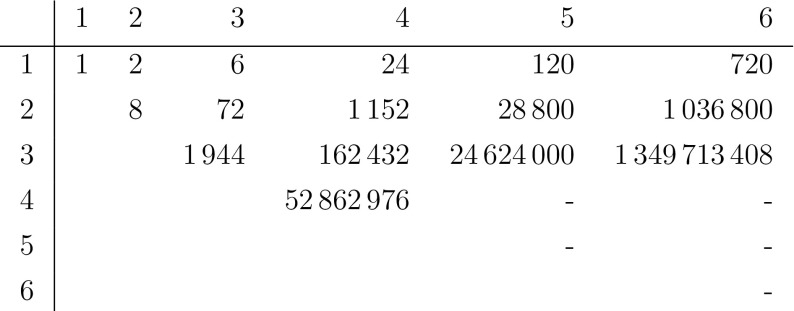
Mixed volumes of the Newton polytopes of (1), numbers in red resp. blue were verified symbolically using Gröbner bases resp. numerically using homotopy continuation (color figure online)

Figure 8 shows a table with the mixed volume for various $(n_1,n_2)$ computed using gfan. We see that the number for $(n_1,1)$ and $(n_1,2)$ corresponds with the theoretical results. Sadly, there is no apparent pattern for $(n_1,n_2)$ with $n_2>2$.

Note that there exist criteria on so-called Newton-degeneracy which guarantee that the mixed volume equals the number of roots (Huber and Sturmfels 1995). However, due to the high dimension of the Newton polytopes, these were infeasible to verify for the cases of interest such as (4, 3).

### Counting solutions using Gröbner bases

Given a zero-dimensional polynomial ideal $I\unlhd \mathbb {C}[x]$, the dimension of $\mathbb {C}[x]/I$ as a $\mathbb {C}$-vector space equals the number of solutions counted with multiplicity, though in our specific case we only have solutions with multiplicity 1. The vector space dimension can be easily read off any Gröbner basis, but computing the Gröbner basis itself is a highly challenging task (Greuel and Pfister 2008, Section 1.8.5). In Fig. 8, red numbers mark all cases for which Gröbner bases were computable in Singular. The respective vector space dimensions (computed using the Singular command vdim) all coincided with the mixed volume. This shows that their coefficients are generic in the context of the BKK Theorem, though for all cases but (3, 3) this was already proven.

### Explicit solutions for (5, 2) and (4, 3)

For the cases (5, 2) and (4, 3), highlighted blue in Fig. 8, we also tried to compute explicit roots using bertini. However, numerical instabilities arose in both cases during the computation, so that the roots computed are most likely incomplete.

For (5, 2) we obtained 28,737 roots, 63 short or $99.8\%$ of the proven 28,800 roots. For (4, 3) we obtained 156,966 roots, 5466 short or $97\%$ of the conjectured 162,432 roots.

## Open questions

We close with three open questions.

### Question 6.1

What is the number of solutions for $(n_{1},n_{2})$?

For binding polynomials of bidegree (*n*, 1) and (*n*, 2), the number of decoupled molecules is given by relatively simple expressions. Assuming that the mixed volumes of the Newton polytopes equals the number of solutions, Table 8 indicates a more complicated pattern in the number of decoupled molecules for (*n*, 3). The smallest interesting example is the case (4, 3) for which we conjecture that the number equals 162432.

### Question 6.2

How many solutions with real, positive values for $g_{i}$ and $w_{i,j}$ exist?

For univariate binding polynomials, the existence of complex roots suggests that the system does strongly interact and cannot be represented by a real decoupled system. In particular it is an indicator for “cooperativity” (Martini et al. 2016). It is neither clear how this concept can be translated to decoupled molecules for two types of ligands nor which characteristic different decoupled molecules share. To develop an understanding, it would be helpful to determine the number of real, positive solutions for small examples.

### Question 6.3

Find an algorithm to compute the minimal interaction energy that a molecule with prescribed binding polynomial has.

For univariate binding polynomials, a quantitative measure for “cooperativity” is mapping the polynomial to the minimal interaction energy which is required to generate it (Martini 2017). In more detail, the *norm* of a molecule is the product of all the *absolutes* of its interaction energies,

$$\begin{aligned} |\mathrm{M}| = \prod |w_{i,j}|, \text { where } |w| := \max \left( w,w^{-1}\right) , \end{aligned}$$

while the *norm* of a polynomial $\Phi$ is the minimal norm of all molecules that give rise to this polynomial. How can we calculate $|\Phi |$? It would be interesting to investigate whether the machinery that has been developed for Euclidean distance degree is applicable in our setting (Draisma et al. 2016).
